# Development and Primary Application of an Indirect ELISA Based on Rep Protein to Analyze Antibodies against Porcine Cocirvirus-like Virus (PCLV)

**DOI:** 10.3390/v14071398

**Published:** 2022-06-27

**Authors:** Zheng Chen, Xifeng Hu, Xiangdong Wu, Yu Li, Zhen Ding, Qinghua Zeng, Tong Wan, Jingyi Yin, Huansheng Wu

**Affiliations:** 1 Department of Preventive Veterinary Medicine, College of Animal Science and Technology, Jiangxi Agricultural University, Zhimin Street, Qingshan Lake, Nanchang 330045, China; chenzheng@jxau.edu.cn (Z.C.); q1159527229@163.com (X.H.); dxywxd2006@126.com (X.W.); yuli12_18@163.com (Y.L.); dingzhenhuz@jxau.edu.cn (Z.D.); linn_zeng@163.com (Q.Z.); jingyiyin2022@163.com (J.Y.); 2 Jiangxi Provincial Key Laboratory for Animal Science and Technology, College of Animal Science and Technology, Jiangxi Agricultural University, Nanchang 330045, China; 3 College of Engineering, Jiangxi Agricultural University, Zhimin Street, Qingshan Lake, Nanchang 330045, China; want0621@163.com

**Keywords:** porcine circovirus-like virus, Rep protein, ELISA, antibody, serum epidemiology

## Abstract

Porcine circovirus-like virus (PCLV) is a member of circovirus that contains a single-strand DNA genome, which may be one of the pathogens that causes diarrheal symptoms in pigs. The Rep protein encoded by the genome of PCLV may be responsible for viral genome replication. The development of serological detection methods for PCLV is of great necessity for clinical diagnosis, as well as epidemiological investigations. Therefore, this study attempted to build an indirect enzyme-linked immunosorbent assay (ELISA) to examine antibodies against PCLV based on the His-tagged recombinant Rep protein. Full-length PCLV Rep protein was induced and expressed in *E. coli* and was purified as an antigen to establish an ELISA detection kit. The purified Rep protein was used to inject into mice to produce specific antibodies. There was no cross-reaction of Rep-based ELISA with antisera against other porcine viruses. The intra-assay and inter-assay coefficient variations (CVs) were 0.644–8.211% and 0.859–7.246%, respectively, indicating good repeatability. The non-cross-reaction with TGEV, PRRSV and PCV2 testing showed high sensitivity and high specificity for this ELISA assay. A total of 1593 serum samples collected from different pig farms in Jiangxi Province were tested for anti-PCLV Rep antibodies, and 284 (17.83%) of the 1593 samples were Rep antibody positive. Altogether, the indirect ELISA detection tool developed in this study could be applied to examine serum of PCLV antibodies with good repeatability, high sensitivity and high specificity. In addition, field sample detection results suggested that the PCLV antibody has a low prevalence in pig populations in Jiangxi Province of China.

## 1. Introduction

To date, Circular Rep-encoding single-stranded DNA (CRESS DNA) viruses have been found in prokaryotic and eukaryotic life worldwide [1,2]. In 1970, the first CRESS DNA viruses were identified [3]. Currently, CRESS DNA viruses contain seven families: *Bacilladnaviridae*, *Smacoviridae*, *Nanoviridae*, *Geminiviridae*, *Redondoviridae*, *Genomoviridae* and *Circoviridae*, some of which have been reported to infect animals, such as pigs, cats and dogs [2,4,5]. The genome of this family virus consists of only one open reading frame (ORF) that encodes the Rep protein [4,6]. Other members of these families contain two ORFs that majorly encode the Rep and Cap proteins. Porcine circovirus-like virus (PCLV) is similar to porcine circovirus (PCV) that has a closed circular, non-enveloped and single-stranded DNA virus [7,8,9,10,11]. However, the gene structure of PCLV is remarkably different from that of PCV, since the genome of PCLV contains only one ORF encoding Rep protein [12]. In 2011, PCLV was first identified in the United States in fecal samples of both healthy and sick pigs via virome sequencing [13]. Recently, PCLV was further identified in Guangxi, Guangdong and Anhui provinces in both piglets and adult pigs with hemorrhagic enteritis [14,15,16]. In addition, PCLV has been reported to be co-infected with other viruses, including porcine epidemic diarrhea virus (PEDV) and porcine circovirus type 2 (PCV2), leading to a great challenge in preventing the transmission of PCLV [15].

Antibody detection can reflect the infection of the virus or not and vaccinations or not [17,18]. Thus, PCLV antibodies can be used to test for PCLV infection or to evaluate the properties of PCLV vaccination, although there are no commercial vaccines against PCLV. Anti-PCLV antibody determination may be more critical than virus detection since antibody titers can reflect the infectious possibility of shedding PCLV and detection of PCLV would be difficult even when using quantitative polymerase chain reaction (qPCR). In addition, compared with antibody detection methods based on whole viruses, recombinant protein expressed protein expressed in *E. coli* and purified, and used for indirect ELISA can reduce its cross-reactivity and thus decrease false positives [19,20]. In this study, the full length of His-tagged Rep protein was used as the coating antigen for the first time to develop an indirect ELISA for detecting anti-PCLV antibodies, since the Rep protein is the only protein of PCLV. Furthermore, we validated the specificity, sensitivity and repeatability of the novel indirect ELISA kit. We believe that our study will provide an important candidate serological diagnostic method for PCLV investigation.

## 2. Materials and Methods

### 2.1. Serum Sample Collection and Viral DNA Extraction

A total of 1593 serum samples were collected from different intensive pig farms located in Nanchang, Shangrao, JiAn, Yichun, Jiujiang, Pingxiang, Yingtan and Ganzhou in Jiangxi Province of China, between January 2021 and December 2021. The Committee of the Ethics of Animal Experiments of Jiangxi Agricultural University supervised serum sample collection. Viral DNA extraction was performed based on the protocols of the TIANamp Virus DNA Isolation Kit (DP315, TIANGEN Biotechnology Co., Ltd., Nanjing, China). The extracted DNA was stored at 4 °C until PCR detection.

### 2.2. Rep Gene Amplification and Vector Construction

To obtain the full-length sequence of the PCLV Rep gene, conventional specific PCR was performed. Briefly, according to the sequence of the PCLV Rep gene uploaded in GenBank (Accession number: MZ960935), a pair of specific premiers was designed: RepF: GCCTCGAGATGCAAAGAGTGCGCGCAAGACGAT; and RepR: GCGAATTCTTATTCGATCATTTGGGGGTATAAG. PCR amplification was performed in a 50 μL reaction volume: 25 μL of 2× Rapid Taq Master Mix (Green Dye Plus, P222-01, Vazyme Biotechnology Co., Ltd., Nanjing, China), 2 μL DNA, 2 μL of each primer (10 μM) and ddH_2_O up to 50 μL. The PCR program was followed: pre-denaturation at 95 °C for 3 min, and subsequently amplified by 35 cycles of 95 °C for 30 s, annealing at 58 °C for 30 s, elongation at 72 °C for 30 s, and a final elongation at 72 °C for 10 min. The purified PCR product was inserted into pUC57 (General Biotechnology (Anhui, China) Co., Ltd.), and the positive clone was sequenced directly by TSINGKE Biotech Beijing Co., Ltd. Next, full length of Rep gene was further sub-cloned into pET32a (+) vector by XhoI and EcoRI enzyme and T4 ligation enzyme to construct the recombinant expression vector, which was ultimately sequenced by General Biotechnology.

### 2.3. His-Rep Protein Purification

Recombinant expression plasmids were transferred into BL21pLyss-competent cells. Moreover, the expression of His-Rep protein was induced in the control of 2.0 mM isopropy1 β-D-1-thiogalactopyranoside (IPTG) at 37 °C for different times (4 h, 6 h, 8 h, 10 h and 12 h). Protein expression was analyzed using 10% SDS-PAGE and, subsequently, Coomassie Brilliant Blue staining. Next, recombinant His-Rep protein was purified with an Ni-NTA affinity chromatography column in the non-denaturing condition, according to the previous literature. Purified His-Rep protein was stored in −80 °C for next usage.

### 2.4. Immunogenicity Determination

Six-week old BALB/C female mice weighing 22 g on average obtained from the Center of Experimental Animal in Jiangxi Traditional Medical University were housed under normal conditions in the animal care facility of Jiangxi Agricultural University. Purified His-Rep protein (40 μg/mice) emulsified using Freund’s complete adjuvant was injected into five different BALB/C mice. After one week, the same mice were injected with the same antigen and Freund’s complete adjuvant. Two weeks later, the mice were immunized using an injection of 200 μg/mice and Freund’s complete adjuvant. One week later, we performed the fourth immunization with an injection of 100 μg/mice and Freund’s complete adjuvant. We collected blood from the cauda artery, which was further separated for serum isolation. At the end of the blood collection, all the mice were euthanized in a CO_2_ chamber. The Rep protein antibody was assessed using the indirect ELISA established in this study. Dilution from 1:1000 to 1:128,000 in PBS was used to analyze the antibody titration.

### 2.5. Indirect ELISA for Detecting Anti-Rep Antibodies

Based on the P/N value, the conditions suitable for indirect ELISA were optimized step by step, including blocking solution, sera dilutions, concentrations of coated protein, HRP-conjugated goat anti-pig IgG and its corresponding reaction times. After the optimization procedure, the following best reaction conditions were obtained: ELISA plate wells were coated with 1.25 μg/mL of purified His-Rep protein. Phosphate-buffered saline tween (PBST) was used to wash the plate three times, then, 100 μL of 1% bovine serum albumin (BSA) was added to each well and incubated at 37 °C for blocking for 1.5 h. After washing three times, 50 μL of 1:800 diluted serum was added to react at 37 °C for 1 h. After washing with PBST three times, 1:10,000 diluted HRP-conjugated goat anti-pig IgG in BSA was further added to react at room temperature (RT) for 1 h. 100 μL of 3,3,5,5-tetramethylbenzidine (TMB) substrate solution was added and incubated at RT for 10 min, which was stopped by adding 100 μL of ELISA Stop Solution buffer. Finally, OD_450_ value was measured.

### 2.6. The Cut-Off Value

The OD_450_ value of each of the 40 negative serum samples was tested three times. The data showed that the standard deviations (SDs) were 0.66 and the average value (χ) was 0.106. The cut-off value of OD_450_ in the conversion was based on the following formula: OD_450_ value = χ+ 3 SDs = 0.304. Finally, the OD_450_ ≥ 0.304 was recorded as a positive sample; if not, it was considered negative.

### 2.7. The Coefficient of Variation Determination

Nine positive serum samples and nine negative serum samples were used to determine intra-assay variation as well as inter-assay variation. The value of each samples’ determination was shown as the mean, standard deviation and coefficient of variation CV (%). Each sample was detected four times using the same methods, which were used to determine the intra-assay CVs. In addition, each sample was analyzed for four different days within the same assay, which was further used to calculate the CVs of the inter-assay.

### 2.8. Sensitivity and Specificity of ELISA test

To determine the sensitivity of this ELISA method, dilutions of immunized mouse serum against Rep protein from 1:50 to 1:6400 were used to detect the Rep protein. The OD_450_ value of 1:6400 was 0.485, which is the closest to the cut-off OD_450_ value, indicating that the 1:6400 dilutions of serum were sensitive for ELISA detection. In addition, to evaluate the specificity of the indirect ELISA, eight serum samples of each against PPRSV, PCV2, TGEV and negative serum against PCLV were used to assess its specificity within the same ELISA method with triplicate experiments, and the OD_450_ value was used to characterize whether the samples were positive or negative.

### 2.9. Field Samples Rep Antibody Detection

A total of 1593 serum samples were collected from intensive pig farms in Nanchang, Shangrao, JiAn, Yichun, Jiujiang, Pingxiang and Ganzhou in Jiangxi Province, China. All serum samples were detected according to the protocol’s indirect ELISA kit. The OD450 value ≥ 0.304 of serum samples was recorded as a positive sample; if not, it was considered a negative sample.

### 2.10. Statistical Analysis

Statistical analyses were performed using SPSS software (SPSS 18.0, Chicago, USA). All data were presented using GraphPad Prism 5.0 software (San Diego, CA, USA). *p* < 0.05 was recorded as statistical significance by *t* test between two groups.

## 3. Results

### 3.1. PCLV Rep Amplification and His-Rep Protein Purification

**Figure 1:**
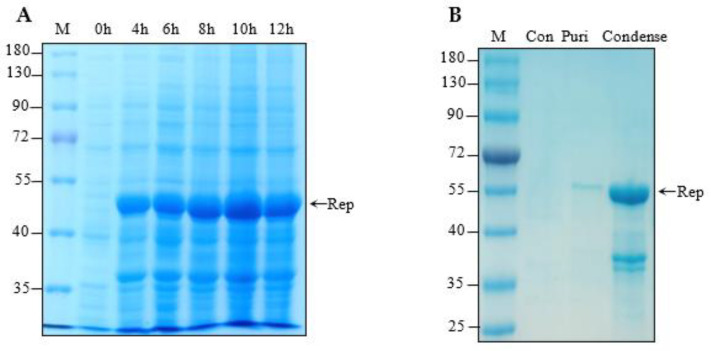
His-Rep protein expression and purification. (**A**) pET-32a-Rep recombinant plasmid transferred into BL21pLyss was induced expression by IPTG at different times (0 h, 4 h, 6 h, 8 h, 10 h and 12 h). Induced protein samples were subjected to SDS-PAGE and Commassie Brilliant Blue staining. (**B**) Expressed His-Rep protein purified by Ni-NTA assay and condensed by ultra filtration, which were further analyzed by SDS-PAGE and Commassie Brilliant Blue staining. Con: control; Puri: purified protein; Condense: condensed protein.

According to previous reports, it was shown that both diarrheic symptoms and healthy pigs were related to PCLV infection [15], and this prompted us to amplify the Rep gene from healthy pigs. To address this, 10 tissues from healthy pigs were selected to amplify the full-length PCLV Rep gene. About 930 bp of the Rep gene was successfully amplified from three of ten tissue samples, which were subsequently inserted into the pET32a vector for induced expression. Sequencing results and multiple sequence alignment showed that the PCLV Rep gene was highly conserved with another PCLV Rep gene accessioned in GenBank, indicating that pET32a-Rep was constructed successfully. The pET32a-Rep recombinant plasmid was transferred into BL21pLyss. The expression of His-Rep was induced by IPTG at different induced times (4 h, 6 h, 8 h, 10 h, 12 h). As shown in Figure 1A, we found that the best expression time was 10 h after IPTG induction within approximately 52 kDa. Meanwhile, there was a specific induced expressed protein with about a 37 kDa molecular mass, which might be expressed by the smaller ORF in the initial sequence of Rep. The recombinant protein was successfully purified by the Ni-NTA method, which was further condensed by ultra filtration (Millipore, MA, USA). The results in Figure 1B reveal that the His-Rep protein was successfully purified with a weak 37 kDa protein (Figure 1B).

### 3.2. Immunogenicity Assessment of PCLV Rep Protein

**Figure 2:**
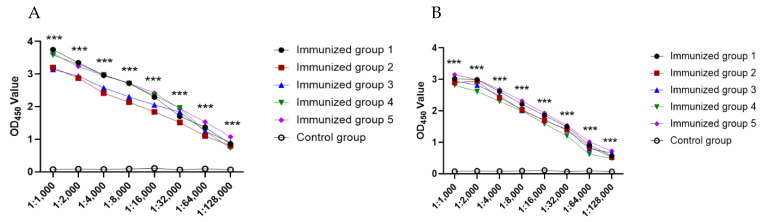
Five mice antibody titers against Rep after (**A**) third around and (**B**) fourth around immunizations. The data were analyzed using SPSS18.0 and visualized by Graphpad Prism software 5.0. OD_450_: optical density 450 nm. *** *p* < 0.001.

Recombinant His-Rep protein accompanied by Freund’s complete adjuvant was injected into five different mice to induce specific antibodies against Rep protein. After the third (Figure 2a) and fourth (Figure 2b) rounds of immunization, serum was collected for detection using the established indirect ELISA. The titers of antibodies in mice were significantly different from those of the control mice.

### 3.3. Cut-Off Value Determination of the ELISA

**Figure 3:**
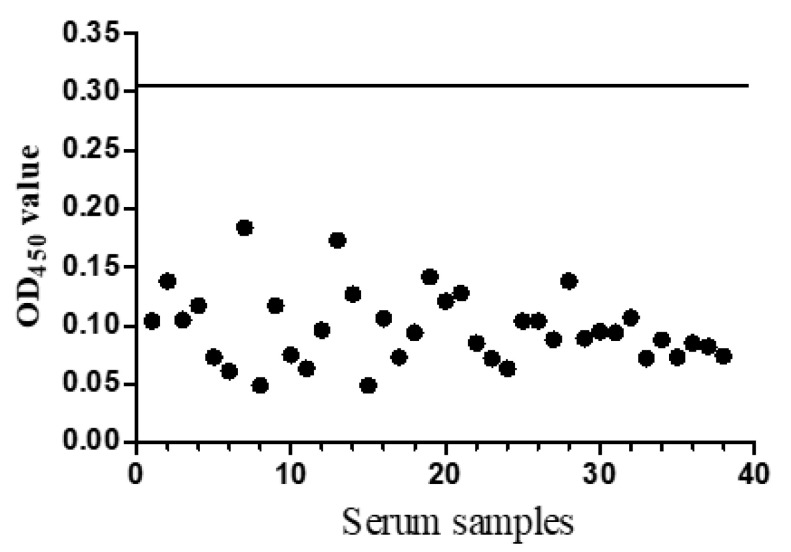
Evaluation of the cut-off OD_450_ value of the indirect ELISA. The black solid line presents the OD_450_ cut-off value (0.304).

Forty negative serum samples were used to characterize the cut-off value of this indirect ELISA. After three independent experiments, the average value (χ) of the negative samples was calculated as χ = 0.016 and standard deviation (SD) was 0.066. The cut-off OD_450_ value was determined using the following formulas: OD_450_ = χ + 3SD = 0.304, indicating that the OD_450_ value ≥ 0.304 of the samples was recoded as positive. If not, the serum was considered negative (Figure 3).

### 3.4. Repeatability of the Indirect ELISA

In general, a well-established indirect ELISA kit requires good working repeatability. To confirm the repeatability of this indirect ELISA kit, 18 serum samples (nine positive and nine negative serum samples) were used to determine its repeatability. The detailed results are shown in Table 1, which revealed that the intra-assay CVs were 0.644–8.221%, while the inter-assay CVs were 0.859–7.246% (Table 1), indicating that this indirect ELISA could work well with excellent repeatability, since a CV% value less than 10% was recorded as good repeatability in general.

### 3.5. Specific Determination of the Indirect ELISA

**Figure 4:**
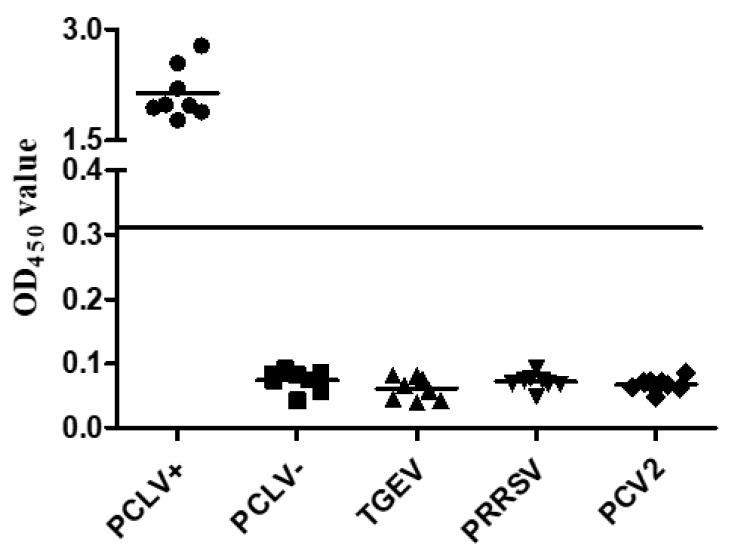
Specificity determination of the indirect ELISA. The black solid line indicates the OD_450_ cut-off value (0.304).

As we all know, good working established indirect ELISA kits must be without non-specific reactions. To address this problem, eight serum samples positive for PCVL Rep and negative for PCLV, TGEV, PRRSV and PCV2 were selected to test the specificity of this indirect ELISA. The results shown in Table 2 and Figure 4 suggest that indirect ELISA cannot cross-react with antisera against other pathogens or PCVL-negative serum samples.

### 3.6. Primary Application of ELISA in Detecting Antibodies

Next, an ELISA kit was applied to detect the antibodies against the PCVL Rep protein. A total of 1593 field serum samples collected from intensive pig farms in Jiangxi Province of China were used to detect the antibodies. The results shown in Table 3 reveal that 17.83% (284/1593) of the samples were positive for antibodies against Rep. Among the serum samples, 26.98% (34/126) of samples from Jiujiang were positive for Rep antibodies, which was higher than that of other pig farms, including Nanchang (17.09%), Shangrao (17.27%), JiAn (13.73%), Yichun (16.83%), Ganzhou (17.69%) and Yingtan (12.18) indicating that the rate of Rep antibody was difference in different areas. Thus, our limited serum samples might not reflect the average Rep antibody distribution in our country, thereby requiring a large-scale investigation of PCLV serum epidemiology.

## 4. Discussion

Porcine circovirus-like virus (PCLV) was first identified in feces samples via a metagenomic-derived virome in both healthy and diarrheic pigs [13]. The full length of the PCLV genome is different, from about 2.7 kb to 4.0 kb [15]. Recently, traditional PCR techniques were used to amplify the full length of the PCLV genome, and it showed that several novel PCLV strains are sequenced in both healthy and diarrheic pigs in Guangdong Province, Anhui Province and Guangxi District, China [14,15]. Although a Taq-Man-based qPCR assay was used to detect PCLV, there is no suitable method for analyzing the antibodies of PCLV [14]. Thus, in this study, we developed an indirect ELISA method to analyze antibodies against PCLV, since the ELISA assay is of low price, has high specificity and is suitable for large-scale detection. To date, only the Rep protein encoded by the circo-DNA genome could be used as the antigen to establish the indirect ELISA kit. Therefore, in this study, the PCLV Rep protein was delivered to the prokaryotic expression system. First, we obtained the full length of the Rep gene and inserted it into the expression vector. After induced expression by IPTG, purified by Ni-NTA and condensed by ultra filtration, SDS-PAGE results revealed that the expression and purification of the His-Rep protein were as effective as expected. Nevertheless, the purification of the His-Rep protein contained a weak, smaller protein that could be detected by His antibody. A smaller Rep protein might be encoded by another ORF in the initial Rep gene, since the genome of PCLV contains five or six potential ORF, and the Rep gene contains two potential ORF [14]. In addition, after the third round, enhanced immunogenicity showed that the specific antibodies produced by injected mice could efficiently react with His-Rep proteins, but not recognize with TGEV, PRRSV, PCV2 and negative PCLV [21,22,23]. An excellent ELISA kit must require good working repeatability, high sensitivity and high specificity [24,25]. To elucidate the properties of this ELISA assay, the repeatability evaluation showed that the values of both the intra-assay and inter-assay were less than 10%, suggesting that this ELISA kit had good repeatability [26,27]. In addition, the results of specificity of this ELISA testing showed that PCLV negative serum or other pathogens’ positive serum, including TGEV, PRRSV and PCV2, did not react.

In general, the diagnosis of virus infectious disease requires antigen (or virus’ nucleotide) detection and antibody detection, both of which can help characterize the prevalence of pathogens [28]. Currently, there is no PCLV vaccine application in China, resulting in the establishment of an ELISA to urgently detect the antibody level of PCLV. In this study, a total of 1593 serum samples from intensive pig farms in Jiangxi Province were collected for field sample detection. The data showed that 17.83% (284/1593) of the samples were PCLV positive, suggesting a low prevalence of PCLV in pigs. However, the positive rate is highly different in different areas, indicating that the prevalence of PCLV might be different all over our country. This phenomenon might be associated with the prevention of another virus, etc. [15].

However, there were several limitations to this study. (1) Since the pathogenicity of PCLV is not clear to date, the serum samples collected were without focalization; therefore, the resulting low positive rate of PCLV antibody cannot reflect the threats of PCLV infection. (2) In general, a new indirect ELISA-based detection kit requires validation by comparison with the same or similar commercial ELISA kit. Nevertheless, although the primary application of this novel ELISA kit has potential use as a diagnostic test, further comparative analysis of this indirect ELISA needs to be performed. (3) Little serum sample numbers from limited areas limited the application of this novel ELISA kit; the low PCLV positive rate in Jiangxi Province did not reflect the positive rate in our country. Hence, large-scale serum antibody investigations need to be further performed in larger areas.

## 5. Conclusions

The established indirect ELISA was with good repeatability, high sensitivity and high specificity for detection of PCLV Rep protein antibody in serum samples. It may have great potential usage for serum epidemiological investigation, as well as the serological diagnosis of PCLV infection. Furthermore, the results revealed a low prevalence of PCLV Rep protein in Jiangxi Province, China, indicating that an extensive serological exploration of the epidemiology of PCLV is necessary in future studies.

## Figures and Tables

**Table 1:** Determination of coefficient of variation (CV) from 18 serum samples.

Sample		Intra-Assay			Inter-Assay		
		χ	SD	CV(%)	χ	SD	CV% (%)
Positive serums	1	1.775	0.016	0.912	1.851	0.072	3.889
	2	1.939	0.034	1.781	1.709	0.039	2.282
	3	1.874	0.023	1.266	1.833	0.035	1.909
	4	1.709	0.026	1.528	1.592	0.029	1.822
	5	1.685	0.016	0.949	1.778	0.018	1.007
	6	1.915	0.012	0.644	1.807	0.019	1.0678
	7	1.805	0.027	1.511	1.698	0.016	0.933
	8	1.807	0.023	1.254	1.735	0.034	1.950
	9	1.844	0.018	0.974	1.813	0.016	0.859
Negative serums	1	0.073	0.003	3.994	0.069	0.005	7.246
	2	0.070	0.005	7.825	0.064	0.002	3.125
	3	0.078	0.003	4.078	0.074	0.002	2.703
	4	0.074	0.004	5.241	0.076	0.005	6.579
	5	0.005	0.064	8.221	0.072	0.002	3.106
	6	0.004	0.068	5.519	0.072	0.003	4.501
	7	0.003	0.078	3.817	0.076	0.003	4.001
	8	0.004	0.069	5.325	0.066	0.003	3.863
	9	0.005	0.076	6.248	0.070	0.002	3.541

**Table 2:** Elucidation of the specificity of this indirect ELISA.

PCLV(+)	Outcome	PCLV(−)	Outcome	TGEV	Outcome	PRRSV	Outcome	PCV2	Outcome
2.784	+	0.083	−	0.045	−	0.069	−	0.062	−
1.982	+	0.058	−	0.042	−	0.074	−	0.071	−
1.774	+	0.092	−	0.075	−	0.049	−	0.063	−
2.205	+	0.043	−	0.081	−	0.068	−	0.048	−
1.976	+	0.074	−	0.040	−	0.093	−	0.085	−
1.885	+	0.084	−	0.058	−	0.077	−	0.072	−
2.548	+	0.086	−	0.066	−	0.074	−	0.071	−
1.942	+	0.075	−	0.082	−	0.069	−	0.066	−

**Table 3:** Rep antibody detection results by ELISA from 1593 serum samples.

	Samples (*n*)	Positive (*n*)	Positive Rate (%)
NanChang	193	33	17.09
ShangRao	411	71	17.27
JiAn	102	14	13.73
YiChun	101	17	16.83
GanZhou	407	72	17.69
Jiujiang	126	34	26.98
Yingtan	353	43	12.18
Total	1593	284	17.83

## Data Availability

All data analyzed in this study are available from corresponding author.

## Abbreviations

Abbreviations	Descriptions
PCLV	Porcine circovirus-like virus
ELISA	enzyme-linked immunosorbent assay
TGEV	Transmissible gastroenteritis virus
PRRSV	Porcine reproductive and respiratory syndrome virus
PCV2	porcine circovirus 2
Rep	Replication protein
PCR	Polymerase chain reaction
CV	coefficient of variation
SDS-PAGE	sodium dodecyl sulfate-polyacrylamide gel electrophoresis
OD	Optical density
Ni-NTA	Nickel NTA Affinity Chromatography Medium
ORF	Open reading frame
